# Bismuth chelate as a contrast agent for X-ray computed tomography

**DOI:** 10.1186/s12951-020-00669-4

**Published:** 2020-08-06

**Authors:** Ji-jun Fu, Jun-jie Guo, Ai-ping Qin, Xi-yong Yu, Qiang Zhang, Xue-ping Lei, Yu-gang Huang, Ming-yue Chen, Jie-xia Li, Yu Zhang, Jing-ping Liu, Yuan-ye Dang, Dan Wu, Xiao-ya Zhao, Zhong-xiao Lin, Yin-lei Lin, Song-pei Li, Ling-yan Zhang

**Affiliations:** 1 The Fifth Affiliated Hospital of Guangzhou Medical University, Guangzhou Medical University, Guangzhou, 510700 Guangdong China; 2 Department of Medical Imaging, The Third Affiliated Hospital, Southern Medical University, Guangzhou, China; 3 The Third School of Clinical Medicine, Southern Medical University, Guangzhou, China; 4 School of Materials Science and Energy Engineering, Foshan University, Foshan, 528000 China; 5 Department of Medical Imaging, Guangzhou First People’s Hospital, School of Medicine, South China University of Technology, Guangzhou, China

**Keywords:** Bismuth agent, DTPA, X-ray computed tomography, Iohexol

## Abstract

**Backgrounds:**

Due to the unexpected side effects of the iodinated contrast agents, novel contrast agents for X-ray computed tomography (CT) imaging are urgently needed. Nanoparticles made by heavy metal elements are often employed, such as gold and bismuth. These nanoparticles have the advantages of long in vivo circulation time and tumor targeted ability. However, due to the long residence time in vivo, these nanoparticles may bring unexpected toxicity and, the preparation methods of these nanoparticles are complicated and time—consuming.

**Methods:**

In this investigation, a small molecular bismuth chelate using diethylenetriaminepentaacetic acid (DPTA) as the chelating agent was proposed to be an ideal CT contrast agent.

**Results:**

The preparation method is easy and cost—effective. Moreover, the bismuth agent show better CT imaging for kidney than iohexol in the aspect of improved CT values. Up to 500 µM, the bismuth agent show negligible toxicity to L02 cells and negligible hemolysis. And, the bismuth agent did not induce detectable morphology changes to the main organs of the mice after intravenously repeated administration at a high dose of 250 mg/kg. The pharmacokinetics of the bismuth agent follows the first—order elimination kinetics and, it has a short half—life time of 0.602 h. The rapid clearance from the body promised its excellent biocompatibility.

**Conclusions:**

This bismuth agent may serve as a potential candidate for developing novel contrast agent for CT imaging in clinical applications.

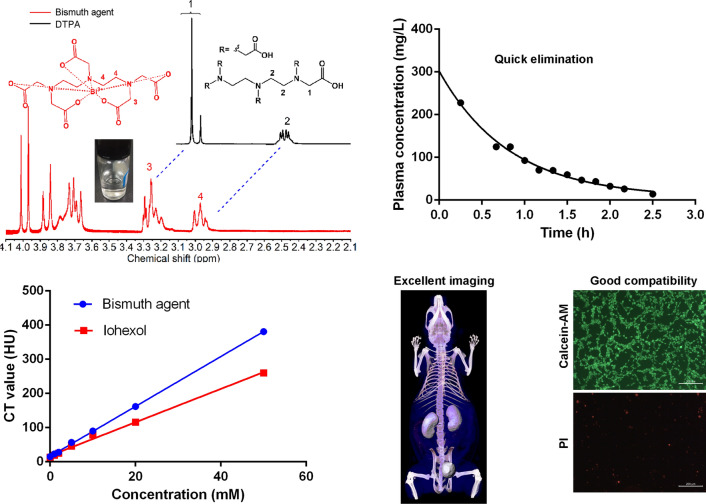

## Background

X-ray computed tomography (CT) produces cross-sectional images of internal organs and structure of the body. Contrast agents (CAs) enhance the visibility of specific regions such as tissue, organs and blood vessels during CT scan. There are two types of CAs currently available for clinical use, small molecules including iodinated contrasts agents (ICAs) as well as newly`developed nanoparticles. These agents are typically administrated intravenously for well-perfused organs or orally for imaging of gastrointestinal organs. In both cases, in addition to emphasize a high performance on imaging, special attention should be paid to the safety of CAs. It is well recognized that lower the dosage of administration is favorable to suppress the toxicity of CAs. Therefore, nanoparticles such as lanthanide nanoparticles [1] and gold nanoparticles [2] are emerging as potential CAs for CT, which can be used at lower dose than traditional CAs and optimized for targeting. However, the high cost and complexity of manufacture have hampered the clinical application of these nanoparticles. Until now, ICAs are still the most frequently used CAs for CT imaging.

There are four types of ICAs for clinical application, ionic monomeric, ionic dimeric, nonionic monomeric and nonionic dimeric [3]. All of them are derivatives of tri-iodobenzoic acid. The overall incidence of side effects induced by ICAs have been suggested to be over 1% [3, 4], including life-threatening adverse event [5]. The side effects induced by ICAs are partially attributed to their hypertonic or hypotonic properties. Although the third generation ICAs, namely nonionic dimer such as iodixanol, possesses iso-osmolality and are believed to induce lower incidence of side effects [6], they still may lead to nephropathy and adverse cardiac events [7–10].

Bismuth preparations are widely used in quadruple therapy for Helicobacter pylori (H. pylori) and the treatment for gastrointestinal disorders such as gastric and duodenal ulcer. A meta-analysis including 35 randomized controlled trails have suggested that no serious side effects were induced by bismuth therapy [11]. In addition, bismuth is one of the most widely used element in nanoparticle contrast agents [12]. Nanoparticles are emerging vesicles for contrast agents with the advantage of long residence time and targeting capacity [12]. However, a cost-effective approach is needed to synthesize nanoparticles in a large scale and its safety needs to be assessed by large scale clinical trials.

In the present study, we constructed a bismuth and diethylenetriaminepentaacetic acid (DTPA) complex and evaluated its potential as CAs for X-ray CT. DTPA has been widely used in lanthanide-based magnetic resonance imaging (MRI) contrast medium, such as Magnevist for the imaging of blood vessels, brain and spin as well as Primovist, a liver-specific CAs. The toxicity and imaging capacity of this bismuth agent was tested in both in vitro and in vivo models. The pharmacokinetics of the bismuth agent was measured in mouse and analyzed by DAS 2.0 software. This study may provide a potential candidate for novel CAs with low toxicity and can be synthesized by cost-effective approaches.

## Methods

### Materials

Bi_2_O_3_, Bi(NO_3_)_3_, DTPA and NaOH were purchased from Aladdin Company (Shanghai, China). The live-dead assay kit and methyl thiazoly tetrazolium (MTT) were purchased from Beyotime Biotechnology (Shanghai, China). Dulbecco’s modified Eagle’s medium (DMEM) was bought from Gibco Corporation.

### Synthesis of the bismuth agent

The bismuth agent was simply prepared by heating Bi_2_O_3_ and DTPA in deionized water. In details, Bi_2_O_3_ (0.5 mmol) and DTPA (1.0 mmol) were added to 10 mL of deionized water, the cloudy mixture was stirred at 80 °C. After about 1 h, the mixture turned from cloudy yellow to clear transparent solution, indicating the end of the reaction. After cooling to room temperature, the solution was filtered through 0.8 µm filter to remove the un-reacted Bi_2_O_3_ or DTPA. The concentration of the bismuth agent was measured by atomic absorption spectrum. Before treating animals, the pH value of the solution should be adjusted to 7.0 by adding NaOH.

### Characterization of the bismuth agent

The bismuth agent without adding NaOH was freeze-dried and used for the following characterizations. The formation of the chelate was confirmed by ultraviolet–visible (UV) spectrum, the Fourier transform infrared spectrum (FTIR), nuclear magnetic resonance spectroscopy (^1^H-NMR), X-ray photoelectron spectroscopy (XPS) and mass spectrum (MS). The bismuth agent and DTPA was dissolved in deionized water and diluted to the concentration of 75 µg/mL, the UV spectrum of both solutions was compared. The bismuth agent and DTPA was dissolved in D_2_O to a final concentration of about 10 mg/mL and, the ^1^H-NMR was measured. To validate the molecular weight of the bismuth agent, ionization mass spectrum (ESI–MS) of the chelate was analyzed.

### The stability of the bismuth agent

After adjusting the pH value of the bismuth agent to 7.0, the solution was stored at 4 °C for 4 months. Before and after the storage, the Nano-Particle Sizing System (Malvern) and transmission electron microscope (TEM) were used to detect any insoluble particles to assess its physical stability. In addition, after the storage, the solution was freeze-dried to get the powder and analyzed by IR spectrum to determine its chemical stability.

### Biocompatibility of the bismuth agent in vitro

The human liver cell line L02 was used to evaluate the in vitro biocompatibility of the bismuth agent. DMEM was used as the media.

Twenty thousand of L02 cells were seeded in 24-well plate and incubated overnight to allow cell adhesion. Then, the bismuth agent was added to the cells to make a final concentration of 500 µM. The cells were allowed to grow for another 24 h before carrying out the live-dead assay according to the kit protocol. Calcein-AM is able to label the living cells and, propidium iodide (PI) stains the nucleus of the dead cells. MTT experiment was also used to measure the viability of the L02 cells. In details, five thousand cells seeded in 96-well plate and incubated overnight to allow cell adhesion. Then, the bismuth agent was added to the cells to make a serial concentrations of 0, 2.5, 6.0, 12.5, 25, 50, 125, 250, 500 µM. After incubation for another 24 h, the viability of the cells was analyzed by MTT method.

### In vitro and in vivo CT imaging of the bismuth agent

The bismuth solutions of a serial concentrations of 0, 1, 2, 5, 10, 20, 50 mM were prepared, their CT values were measured by CT instrument (Philips, 120 kV, 100 mA). The commercially available iohexol was used as a control. The CT values of the diluted iohexol solutions with the same concentrations of iodine element were also measured and compared to those of the bismuth agent.

In this study, KM mice were used in all the animal experiments. The mice were given free access to food and water throughout the experiment. The animal experiments were approved by the ethic committee of Guangzhou Medical University.

The bismuth agent or iohexol was intravenously administered to the mice at the dose of 250 mg/kg. The CT images were obtained immediately after the administration and at the time points of 45, 90, 120 min (Aloka, Japan, 80 kV, 40 µA). The CT values of the organs at different time points were measured and, 3D images were also constructed.

### In vivo distribution and pharmacokinetics of the bismuth agent

To analyze the in vivo distribution of the bismuth agent, the chelate was intravenously administered to the mice at the dose of 250 mg/kg. Two hours after the administration, the mice were sacrificed and the main organs of heart, liver, spleen, lung and kidney were dissolved in concentrated nitric acid at 70 °C for 2 h. Then, the samples were diluted by deionized water and, the bismuth element concentration was quantified by atomic absorption spectrum (AAS).

To investigate the pharmacokinetics of the bismuth agent, the chelate was intravenously administered to the mice at the dose of 250 mg/kg. Before the administration and 15, 40, 50, 60, 70, 80, 90, 100, 110, 120, 130, 150 min after the administration, 10 µL of the blood was collected from the tail and dissolved in concentrated nitric acid. Then, the samples were diluted by deionized water and, the bismuth element concentration was quantified by AAS. The blood concentrations were analyzed by DAS 2.0 software to study the pharmacokinetic parameters of the bismuth agent, including the elimination rate constant (k), initial plasma concentration (C_0_), the half-life time (t_1/2_), the apparent distribution volume (V), the clearance (CL) and the area under the curve (AUC_0→∞_).

The standard samples of the bismuth element were prepared by dissolving Bi(NO_3_)_3_ in 10% nitric acid solution and diluted by deionized water to get a serial concentrations of 0, 0.5, 1, 2, 5, 10, 20 ppm.

### Hemolytic test and the in vivo biocompatibility

Mouse blood was collected and centrifugated at 800 rpm to get the red blood cells (RBCs). Then, the RBCs were washed by saline twice. Then, 900 µL of the bismuth agent solutions with saline as the solvent (after pH adjustment) was added to 100 µL of the RBC sample to get a serial concentrations of 10, 20, 50, 100, 200, 500, 1000 µM. The RBCs treated by deionized water or saline was used as the positive or negative control, respectively. The samples were left undisturbed for 24 h, and then, were centrifugated to discard the cells and, the visible absorption at 540 nm of the supernatant fluid was measured.

To evaluate the in vivo biocompatibility of the bismuth agent, the mice were intravenously treated by the chelate at 250 mg/kg at day 0, 2, 4, 6. At day 7, the mice were sacrificed and the main organs of heart, liver, spleen, lung and kidney were sliced and H & E stained. Morphology of the main organs were pictured and analyzed to evaluate the systemic toxicity of the chelate.

### Statistic analysis

All values were expressed as mean ± standard deviation (SD). All comparisons were performed by the two-tailed Student's *t* test. A *p* value less than 0.05 was taken as statistically significant.

## Results

### Preparation and identification of bismuth-based contrast agent

**Fig. 1:**
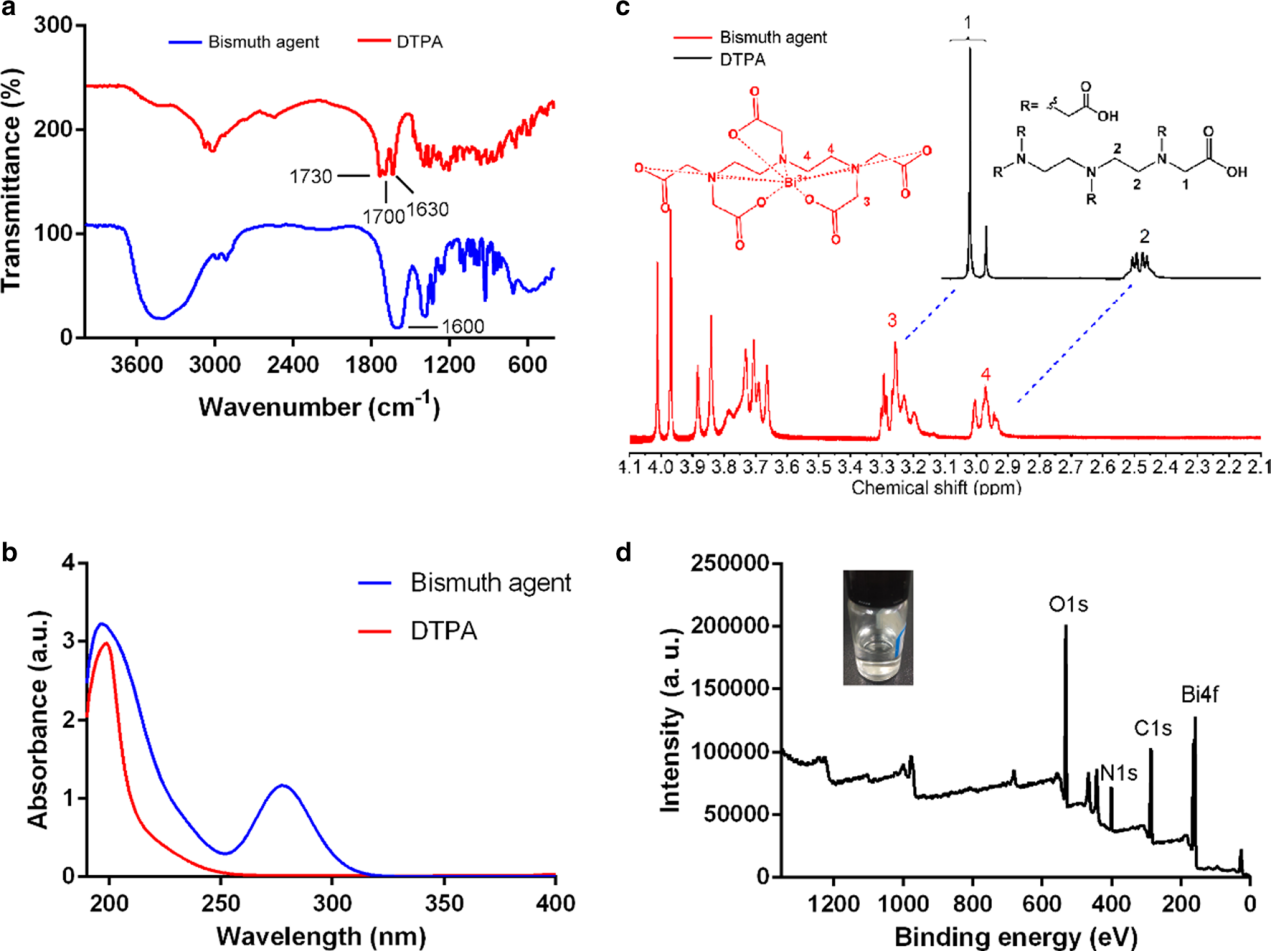
The (**a**) FTIR spectrum, (**b**) UV spectrum and (**c**) ^1^H-NMR spectrum of the bismuth agent and DTPA, (**d**) XPS spectrum of the bismuth agent (insert: the picture of bismuth agent in solution (pH 7.0) after storage at 4 °C for 4 months)

The bismuth agent was prepared simply by heating Bi_2_O_3_ with DTPA at 85 °C in deionized water. The formation of the chelate was identified by FTIR, UV and ^1^H NMR spectrum. In Fig. 1a, DTPA alone showed vibration bands at 1730, 1700 and 1630 cm^−1^, indicating the presence of C = O in carboxyl group. After the chelation with bismuth, a single peak at 1600 cm^−1^ was observed, indicating the O-C-O stretch. The results confirmed the coordination between carboxyl group and Bi^3+^. As shown in the UV spectrum (Fig. 1b), DTPA had a single peak absorption at approximately 200 nm. The bismuth agent showed an additional absorption peak at approximately 278 nm. The formation of the chelate was further identified by ^1^H NMR spectrum (Fig. 1c). After the chelation with bismuth, a singlet and multiplet representing CH_2_ were replaced by complicated multiplets in ^1^H NMR spectrum, suggesting the formation of chelate with bismuth. The XPS spectrum in Fig. 1d further indicated the successful chelation of Bi element with DTPA. In the mass spectra (Additional file 1. Fig. S1), the m/z peak at 600.1000 stands for [M + H] of the compound and, matches well with the theoretical molecular weight of the bismuth agent.

After storage of the bismuth agent (pH7.0) at 4 °C for 4 months, the solution was transparent (Fig. 1d insert) and, insoluble particles were not detectable by TEM and dynamic light scattering (DLS) method. The result implied the good physical stability of the bismuth agent. The bismuth agent (pH7.0) after storage displayed similar IR spectrum (Additional file 1. Fig. S2) to the newly prepared agent (Fig. 1a), with the typical single peak at 1600 cm^−1^. The result indicated the excellent chemical stability of the bismuth agent.

### Toxicity and performance on CT imaging in vitro

**Fig. 2:**
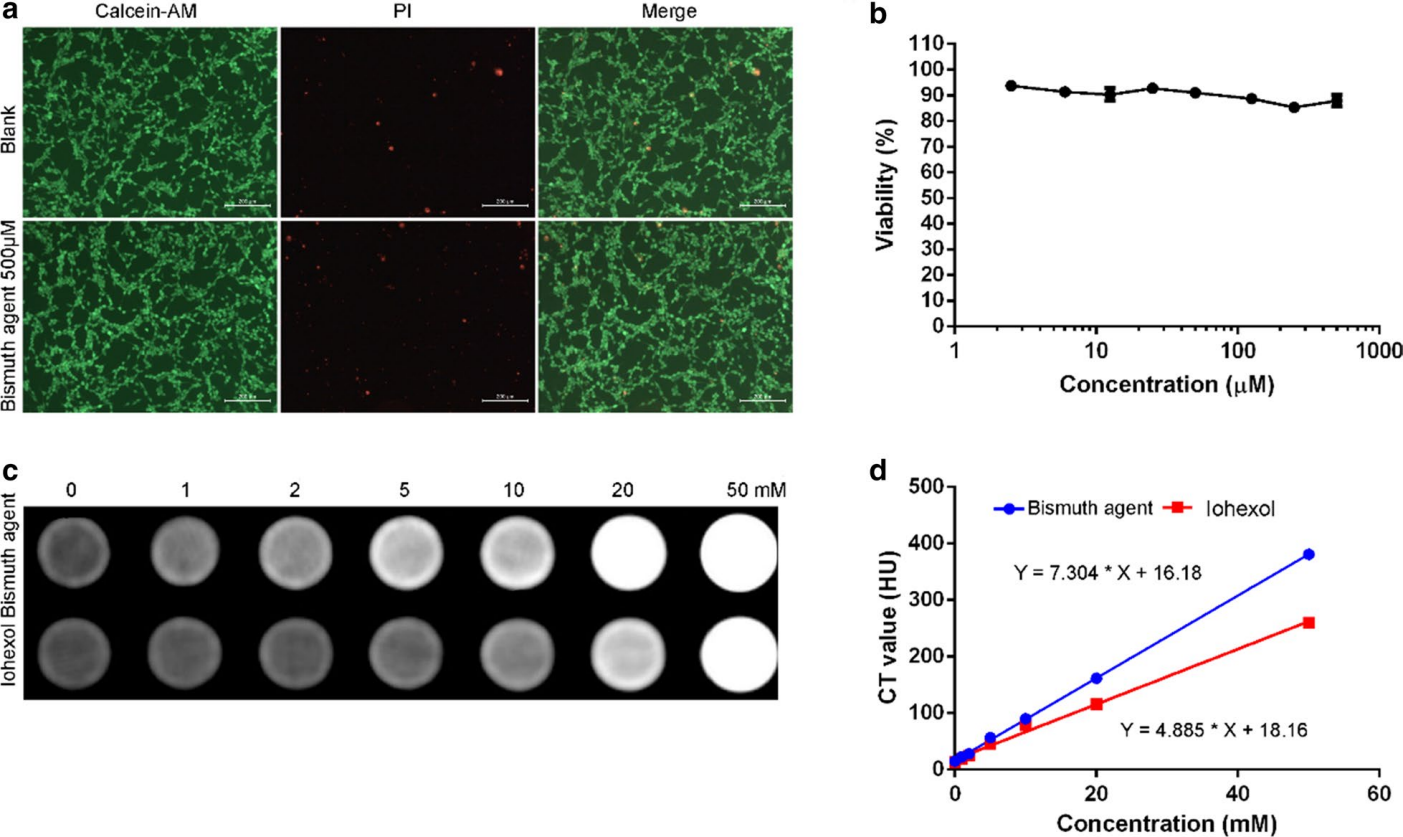
**a** The live—dead assay and (**b**) MTT test of the L02 cells treated by the bismuth agent; **c** The CT images and (**d**) the CT values of the bismuth agent and iohexol with different concentrations

The toxicity of synthesized bismuth agent was firstly evaluated by using calcein-AM and PI to stain live and dead cells, respectively. As shown in Fig. 2a, there is no indication of increased number of dead cells when bismuth agent was added to L02 cells at the concentration of 500 μM for 24 h, the cell morphology and viability was similar to the control group. The toxicity was further assessed by MTT method (Fig. 2b). The results showed that bismuth agent did not induce apparent reduction in cell viability up to the concentration of 500 μM. Both results confirmed excellent biocompatibility of the bismuth agent.

The performance of bismuth agent on CT imaging were evaluated in vitro. As shown in Fig. 2c, compared with iohexol, bismuth chelation showed significantly enhanced brightness on imaging at the serial concentrations of 1, 2, 5, 10, and 20 mM. Figure 2d showed a linear regression between CT value and the agent concentration. Both bismuth agent and iohexol obtained a R value bigger than 0.99, indicating a linear dose–response relationship. However, bismuth chelation showed a greater value of slop, indicating a stronger capacity for CT imaging than iohexol. These results indicated that the synthesized bismuth chelate possesses satisfying biocompatibility and superior capacity in CT imaging in vitro.

### Performance on CT imaging in vivo

**Fig. 3:**
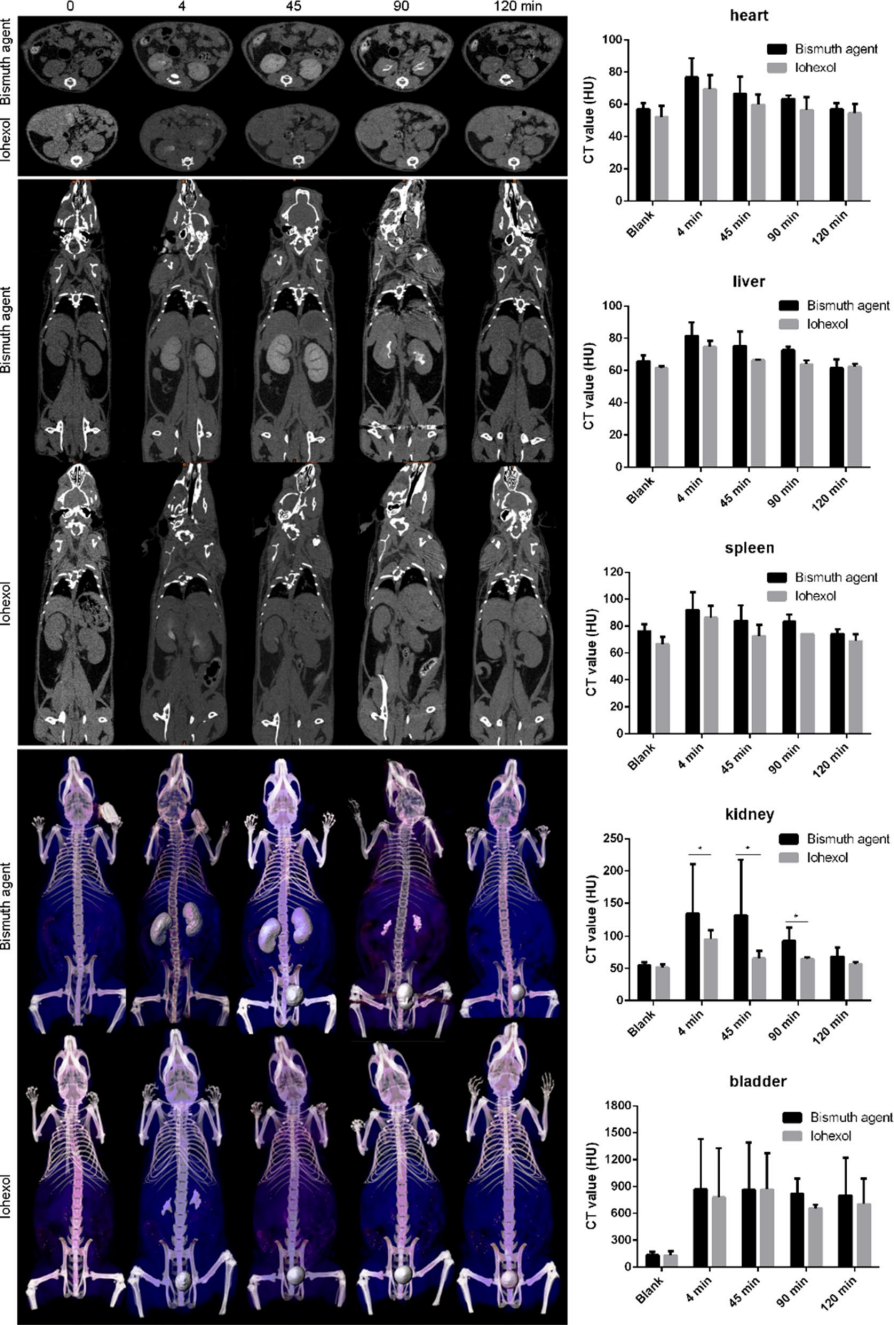
The 2D, 3D CT images and the corresponding CT values of the bismuth agent or iohexol intravenously treated mouse (*Means statistic difference, *p* < 0.05)

The bismuth agent was administrated to mice by *i.v.* injection for CT imaging in vivo. As shown in 2D and 3D CT images in Fig. 3, bismuth chelation enhanced the imaging contrast of CT for kidney at 4 and 45 min after the injection, whereas iohexol provide obscured kidney image in the same experimental setting. The CT values of different organs were quantified in Fig. 3, compared to the iohexol, bismuth agent achieved stronger signal for kidney detection at 4, 45 and 90 min after administration. At 120 min, the effects disappeared and the signal strength of bismuth chelation group was comparable to the blank. Iohexol induced momentary signal improvement in the kidney only in the first few minutes and, the strength decreased quickly to the base line at 45 min. In regard of the bladder, both bismuth chelation and iohexol induced strong contrast in CT imaging, suggesting they are eliminated through urine. Despite slight enhancement, there were no significant difference between bismuth chelation and iohexol in CT imaging for other tissues including heart, liver and spleen. Above results suggested that the synthesized bismuth chelate achieved better performance for kidney CT imaging than iohexol in mouse.

### Pharmacokinetics, in vivo distribution and toxicity of bismuth contrast agents

**Fig. 4:**
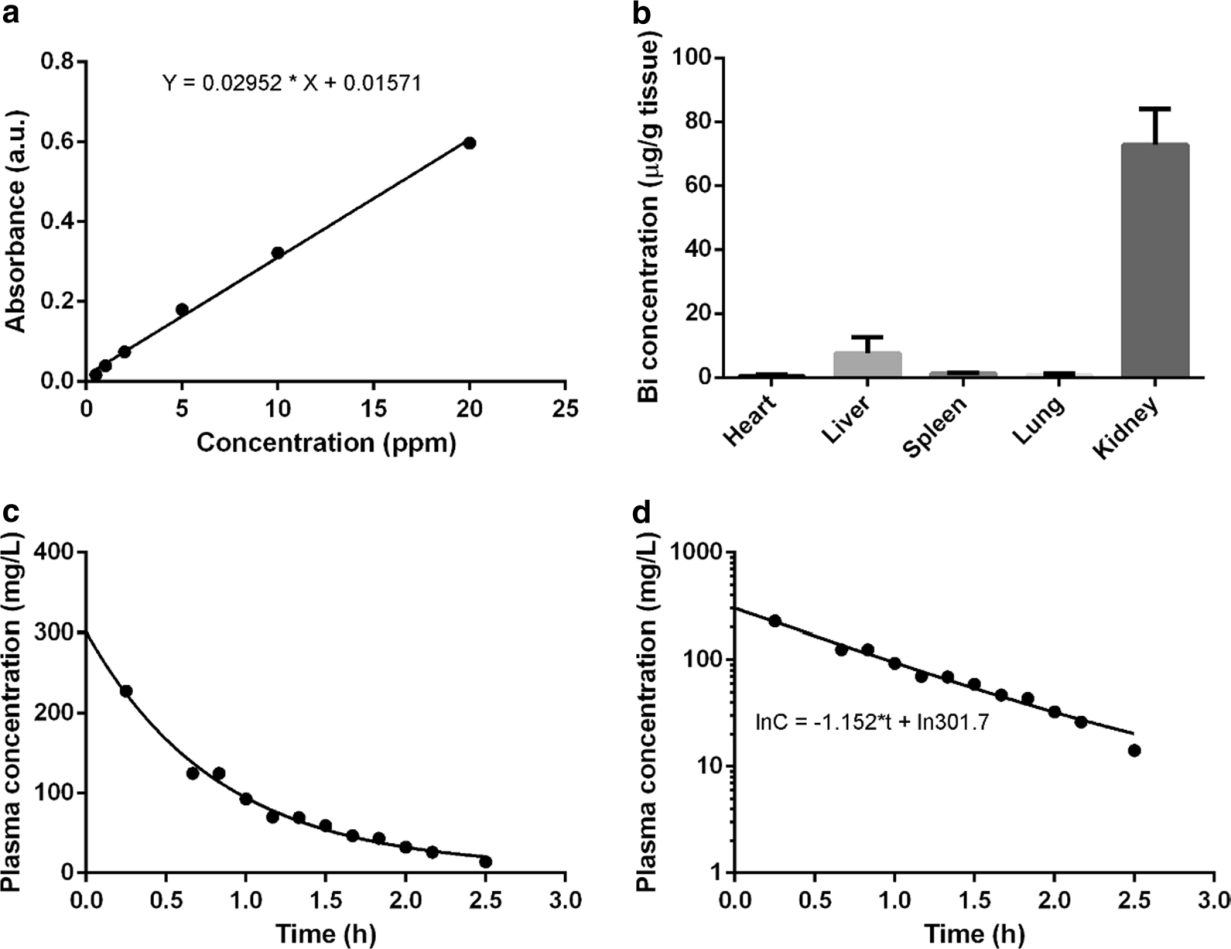
**a** The standard curve of the Bi element measured by AAS; **b** The biodistribution of the bismuth agent in mice; **c** The blood drug concentration—time curve of the intravenously administered bismuth agent; **d** The natural log of blood drug concentrations plotted with time

In order to measure pharmacokinetic parameters and in vivo distribution for the synthesized bismuth chelate, a standard curve was constructed for the detection of bismuth using atomic absorption spectrometry (Fig. 4a). Regression analysis revealed the coefficient of determination R^2^ = 0.9971 for linear regression. Pharmacokinetic properties were measured by administrating bismuth chelation to mice through *i.v.* injection. Bismuth distribution was determined in tissues including heart, liver, spleen, lung and kidney, of which kidney showed the highest concentration of bismuth (Fig. 4b). This is consistent with the results of CT imaging in vivo. The plasma concentration was monitored as shown in Fig. 4c and a non-linear relationship with time was observed. When the natural log of plasma concentrations were plotted with time, an approximately linear regression was obtained (Fig. 4d). These results indicated that the elimination of synthesized bismuth chelation followed the first-order elimination kinetics. Pharmacokinetic parameters were calculated by DAS and a Akaike’s Information Criterion (AIC) value of − 20.927 were obtained for one-compartment model. The elimination rate constant (k), initial concentration (C_0_) and half-life time (t_1/2_) were calculated to be 1.152 h^−1^, 301.7 mg/L and 0.602 h respectively. The apparent distribution volume (V) was calculated to be 0.617 L/kg by dividing dosage by initial plasma concentration. The clearance (CL) was calculated to be 0.710 L/(kg*h). Area under the curve (AUC_0→∞_) was estimated to be 351.917 h*mg/L. The short half-life time indicated that bismuth agent could be quickly excreted, which is a basic requirement for CAs from the perspective of acceptable toxicity.

**Fig. 5:**
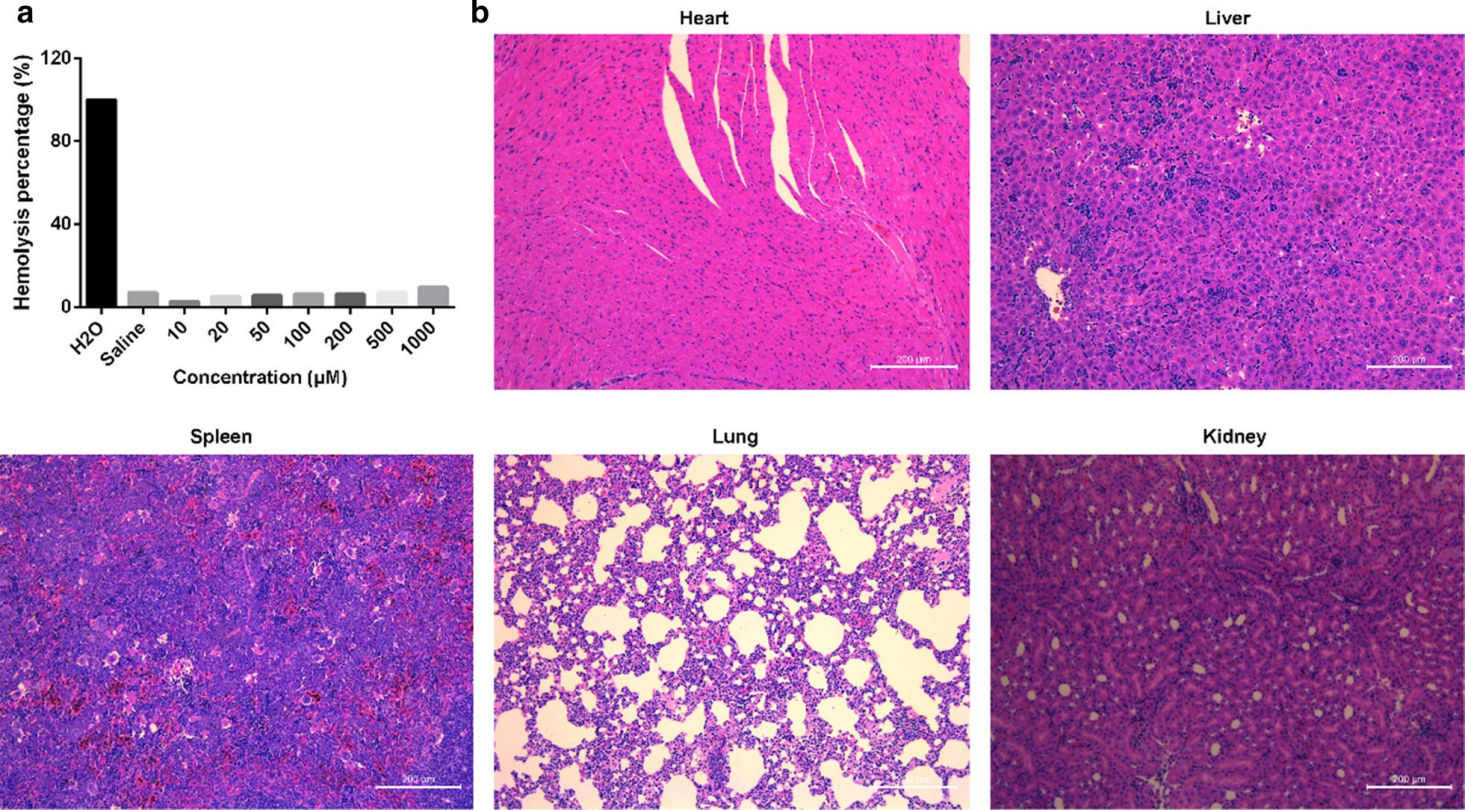
**a** The hemolysis test of the bismuth agent; **b** The H & E images of the main organs of the bismuth agent repeated treated mice

The effect of bismuth agent on hemolysis was tested in a series of concentrations from 10 to 1000 μM with water as a positive control and saline as a negative control. The results showed that there was no obvious hemolysis induced by bismuth chelation up to the concentration of 1000 μM (Fig. 5a). Tissues were collected from mice that administrated with bismuth chelate and H&E staining were performed (Fig. 5b). There was no apparent tissue damage in the heart, liver, kidney, lung and spleen. The results confirmed the good biocompatibility and negligible toxicity of the bismuth agent in vivo.

## Discussion

In the present study, a bismuth chelate was synthesized and its potential application on X-ray computed tomography was evaluated. This bismuth agent showed no apparent toxicity in L02 cells and did not induce morphological changes of organs such as heart, liver, kidney, lung and spleen. The imaging capacity of bismuth agent is superior to iohexol as indicated by higher CT values in vitro as well as in vivo for the imaging of kidney. The plasma clearance of bismuth chelation followed the first-order elimination kinetics with a short half-life time of 0.602 h, which guaranteed a short residence time in vivo and its biosafety.

Although the third generation ICAs possesses iso-osmolality and induce lower incidence of side effects, they still may lead to nephropathy and adverse cardiac events [10, 13]. A clinical trial including 475 patients with myocardial infarction who undergo primary percutaneous coronary intervention compared the side effect of the second generation low-osmolar contrast medium iopromide and the third generation iso-osmolar contrast medium iodixanol on acute kidney injury and myocardial infarction [10]. The results of this study showed that both iopromide and iodixanol induced more the 10% incidence of aggravation of kidney injury and increases of the risk of myocardial infarction. Furthermore, these 2 contrast media all lead to over 5% major adverse cardiac events including cardiac death, reinfarction, and rehospitalization for heart failure within 1 month. These clinical evidence indicates that the discovery of new contrast agent with low toxicity and side effects is desperately needed.

Nanoparticles are emerging as a potential candidate for the CAs with long blood circulation time and tumor-targeting capacity. Bismuth has similar imaging capacity to gold, however it is much less expensive. It has been shown that PLGA nanoparticles encapsulating bismuth nanocrystals and coumarin-6 can be used for CT and fluorescence imaging in vitro [14]. Bismuth and dodecanethiol nanoparticles were synthesized for CT imaging of gastrointestinal (GI) tract [15]. It has been suggested that bismuth nanoparticles may have synergistic effects on photothermal therapy and thermoradiotherapy. It has been shown that thiol-capped bismuth nanoparticles encapsulated by PEGylated phospholipids accumulated in tumor tissue and facilitated tumor CT imaging and thermoradiotherapy in mice [16]. Bismuth sulfide nanorods has been shown to visualized tumor and provide photothermal effects for tumor suppression in mice [17]. Although bismuth nanoparticles enhance contrast for CT imaging, their manufacture is complicated and time-consuming compared with the synthesized bismuth chelate in this study. Furthermore, the long circulation time of nanoparticles may increase the risk of side effects. However, further investigation is needed to evaluate the performance of the synthesized bismuth chelate in photothermal and radiation therapy.

The synthesized bismuth contrast agent showed low toxicity both in vitro and in vivo. Our results indicated that its imaging capacity was superior to iohexol. This allows us to adopt lower concentration of bismuth contrast agent to achieve the same strength of imaging. Lower the dose of contrast media may facilitate the reduction of toxicity. Pharmacokinetic studies indicated that the plasma clearance of this bismuth contrast agent followed first-order elimination kinetics, suggesting that the elimination rate of bismuth contrast agent is directly proportional to its plasma concentration. This reduces the risk of toxic effects that may induced by increasing the dose of drugs that followed zero-order kinetics or non-linear model. Furthermore, the limited retention time of bismuth chelate, which is indicated by a short half-life time of 0.602 h, may also reduce the risk of side effects. Therefore, this bismuth chelate may serve as contrast agent for CT imaging.

## Conclusions

In this study, bismuth agent was prepared for CT imaging by simply using DTPA as chelating agent. The preparation method is cost-effective and time-saving. The bismuth agent is superior to iohexol in the aspect of CT imaging of kidney in mice. In addition, the bismuth agent possesses low toxicity both in vivo and in vitro. Its plasma clearance followed the first-order elimination kinetics with a short half-life time of 0.602 h, which means short residence time in vivo and improved biosafety. The bismuth agent was supposed to overcome the drawbacks of the clinical ICAs and bring better imaging performance. Although further clinical studies are needed, this bismuth chelate is a clinically potential candidate as CAs for CT.

## Supplementary information

**Additional file 1: Figure S1.** +ESI-MS spectrum of the bismuth chelate. **Figure S2.** The IR spectrum of bismuth agent in solution (pH 7.0) after storage at 4 °C for 4 months.

## Data Availability

All data used to generate these results is available in the main text.

## Abbreviations

CT: Computed tomography
DTPA: Diethylenetriaminepentaacetic acid
CAs: Contrast agents
ICAs: Iodinated contrasts agents
MRI: Magnetic resonance imaging
TEM: Transmission electron microscope
DLS: Dynamic light scatering
MTT: Methyl thiazoly tetrazolium
DMEM: Dulbecco’s modified Eagle’s medium
UV: Ultraviolet–visible
FTIR: Fourier transform infrared spectrum
ESI–MS: Ionization mass spectrum
PI: Propidium iodide
AAS: Atomic absorption spectrum
k: Elimination rate constant
C_0_: Initial plasma concentration
t_1/2_: Half-life time
V: Apparent distribution volume
CL: Clearance
AUC_0→∞_: Area under the curve
RBCs: Red blood cells
