# Supplementary material for: Bismuth chelate as a contrast agent for X-ray computed tomography
Source: J Nanobiotechnology. 2020 Aug 6;18:110. doi: 10.1186/s12951-020-00669-4 (PMC7412782; doi:10.1186/s12951-020-00669-4)
Supplement: Supplementary file 1 — Additional file 1: Figure S1. +ESI-MS spectrum of the bismuth chelate. Figure S2. The IR spectrum of bismuth agent in solution (pH 7.0) after storage at 4 °C for 4 months. [file 12951_2020_669_MOESM1_ESM.docx]

Fig. S1 +ESI-MS spectrum of the bismuth chelate





Fig. S2 The IR spectrum of bismuth agent in solution (pH 7.0) after storage at 4 °C for 4 months.
